# Impact of Hydrogen Bonds Limited Dipolar Disorder in High-k Polymer Gate Dielectric on Charge Carrier Transport in OFET

**DOI:** 10.3390/polym12040826

**Published:** 2020-04-05

**Authors:** Bartosz Paruzel, Jiří Pfleger, Jiří Brus, Miroslav Menšík, Francesco Piana, Udit Acharya

**Affiliations:** Institute of Macromolecular Chemistry of the Czech Academy of Sciences, Heyrovsky Sq. 2, 162 06 Prague, Czech Republic; pfleger@imc.cas.cz (J.P.); brus@imc.cas.cz (J.B.); mensik@imc.cas.cz (M.M.); piana@imc.cas.cz (F.P.); acharya@imc.cas.cz (U.A.)

**Keywords:** OFET, polar dielectric, hydrogen bond, dipolar disorder, energy disorder, charge carrier mobility

## Abstract

The paper contributes to the characterization and understanding the mutual interactions of the polar polymer gate dielectric and organic semiconductor in organic field effect transistors (OFETs). It has been shown on the example of cyanoethylated polyvinylalcohol (CEPVA), the high-k dielectric containing strong polar side groups, that the conditions during dielectric layer solidification can significantly affect the charge transport in the semiconductor layer. In contrast to the previous literature we attributed the reduced mobility to the broader distribution of the semiconductor density of states (DOS) due to a significant dipolar disorder in the dielectric layer. The combination of infrared (IR), solid-state nuclear magnetic resonance (NMR) and broadband dielectric (BDS) spectroscopy confirmed the presence of a rigid hydrogen bonds network in the CEPVA polymer. The formation of such network limits the dipolar disorder in the dielectric layer and leads to a significantly narrowed distribution of the density of states (DOS) and, hence, to the higher charge carrier mobility in the OFET active channel made of 6,13-bis(triisopropylsilylethynyl)pentacene. The low temperature drying process of CEPVA dielectric results in the decreased energy disorder of transport states in the adjacent semiconductor layer, which is then similar as in OFETs equipped with the much less polar poly(4-vinylphenol) (PVP). Breaking hydrogen bonds at temperatures around 50 °C results in the gradual disintegration of the stabilizing network and deterioration of the charge transport due to a broader distribution of DOS.

## 1. Introduction

The idea of “organic electronics” is supported by the actual need of the production of low-end electronic devices using large area printing technology. Organic field effect transistors (OFETs) are the basic electronic components in various flexible organic complementary circuits needed for the majority of applications. Most of the OFETs available today, however, still suffer from low charge carrier mobility and not sufficient stability and reproducibility of their electrical parameters. For this reason, significant attention is put on the development of new organic semiconducting materials. Currently, a large number of organic materials are available, which can be roughly divided into three main groups: i) highly crystalline low-molecular weight materials like benzothienobenzothiophene (Cn–BTBT), dinaphthothienothiophene (Cn-DNTT) or dibenzothiophenothienothiophene (DBTTT) derivatives, having the charge carrier mobility around 2 cm^2^ V^−1^ s^−1^, 12 cm^2^ V^−1^ s^−1^ and 14 cm^2^ V^−1^ s^−1^, respectively [1,2,3,4,5,6], ii) semicrystalline conjugated polymers with reported mobility up to 1.1 cm^2^ V^−1^ s^−1^ as in the case of thiophene–thienothiophene copolymer PBTTT [7,8] and iii) donor–acceptor (D–A) structures, considered as the most promising materials now, reaching the mobility higher than 10 cm^2^ V^−1^ s^−1^ [9,10,11,12]. However, regardless of the organic materials used, OFETs often exhibit deviations from ideal electrical characteristics, which make their application in electronic circuitry difficult. Such deviations even complicate a reliable extraction of basic transistor parameters such as charge carrier mobility or threshold voltage. As analyzed in the excellent reviews by Braga and Horowitz [13], Sirringhaus [14] and Phan et al. [15], those deviations from the ideal transistor behavior are mostly expressed as a hysteresis in the transfer current–voltage characteristics, which can show either a decreasing (downward curvature) or an increasing (upward curvature) slope with the increasing gate voltage. It is widely accepted that the hysteresis with a back sweep current higher compared to the forward direction, often observed in OFETs with polymeric gate dielectrics such as poly(4-vinylphenol) (PVP) or polyvinylalcohol (PVA), arises from a slow polarization of the strongly polar groups. Contrary, the lower back sweep current is assigned to trapping of free charges at the dielectric–semiconductor interface or in the organic semiconductor itself [16,17,18,19]. A nonlinearity in the plot of the square root of transfer characteristics is also frequently observed. When this nonlinearity has the character of a rising slope at higher gate voltage it is usually attributed to the filling of more localized states at the tail of the density of states distribution (DOS) with increasing voltage. In such a case the charge transport then takes place through more delocalized states with higher energy [20,21,22].

The properties of the dielectric material used in the gate have a direct impact on the above mentioned current–voltage non-idealities and on the threshold voltage of the OFET. For example, it is well known that various functional groups located at the surface of the dielectric could serve as trapping centers for the charge transport in the semiconductor close to the interface with the dielectric [23]. It has been also shown by Salleo et al. that the charge carrier trapping in the semiconductor layer is correlated with the dielectric constant of the gate insulator [24]. Veres et al. compared electrical characteristics of OFETs with an amorphous polymer in the active channel combined with various polymeric dielectrics and found that the highest mobility was obtained for low-k dielectrics [25]. Higher polarity of the dielectrics leads to a broadening of the DOS of the semiconductor in the vicinity of the semiconductor/insulator interface due to the static dipolar disorder, which in turn leads to the lower charge carrier mobility. Later, Richards et al. proposed a model, which shows that the semiconductor DOS is broadened significantly in the first few angstroms from the interface with the dielectric, resulting in an increase of the density of deeper trapping states [26]. These findings are in agreement with the results reported by Veres et al., however, the authors pointed out one exception, namely cyanopullulan, which did not fit to the overall observed trends.

In 2011 Xu et al. investigated OFETs based on high-k cyanoethyl pullulan (CEP) polymer gate dielectric and reported observations of quasi-ordering of spontaneously associated highly polar surface nitrile groups. Breaking these associations at higher temperature during the drying stage resulted in a dramatic decrease of the charge carrier mobility, which was attributed to a larger molecular disorder in the adjacent pentacene layer [27,28].

Inspired by the above mentioned literature, in our work we investigated OFETs with another high-k gate dielectric, namely cyanoethylated polyvinyl alcohol (CEPVA). CEPVA is an amorphous polymer containing highly polar side groups. Its glass transition temperature, T_g_, is around room temperature. Similar to Xu et al. and Veres et al., we observed that the charge carrier mobility depends on the drying temperature of the gate dielectric layer. By means of IR and NMR spectroscopy we correlated this behavior with a formation of a physical network stabilized by hydrogen bonds. However, in contrast to the Xu et al. we attributed the reduced mobility to the broader distribution of the semiconductor DOS due to significant dipolar disorder in the dielectric layer, which in turn depends on the drying conditions of the CEPVA dielectric layer. The electrical characteristics of the OFETs are discussed in terms of charge transport controlled by the 1D drift-diffusion in a semiconductor with DOS distribution influenced by hydrogen bond limited dipolar disorder. Moreover, we show that changes in the dipolar disorder due to the dissociation of hydrogen bonds could, in some cases, overwhelm the temperature dependence of the thermally activated charge transport and result in a misinterpretation of the negative coefficient, dµ/dT < 0. 

## 2. Materials and Methods

### 2.1. Materials

Poly(4-vinylphenol) (PVP, Mw = 25,000), (hydroxymethyl)benzoguanamine (HMBG—crosslinking agent), propylene glycol monomethyl ether acetate (PGMEA) 99.5%, purity grade, N, N-dimethylformamide (DMF) anhydrous 99.8% purity grade and toluene CHROMASOLV HPLC 99.9%, purity grade, all obtained from Sigma Aldrich, were used as received. 6,13-Bis(triisopropylsilylethynyl)pentacene (TIPS-P), and the ultra-flat glass substrates coated with a 20 nm layer of synthetic quartz were purchased from Ossila Ltd (Sheffield, UK). Cyanoethylated polyvinyl alcohol (CEPVA) was synthesized in the laboratories Joint Stock Co. Plastpolymer (St-Petersburg, Russia) via substitution of polyvinyl alcohol (PVA) with the following characteristics: density ρ ~1.2 g cm^−1^, –CN substitution 75% (evaluated by elemental analysis), M_p_ = 1.37 × 10^5^, M_n_ = 7.95 × 10^5^ and PD = 1.67.

### 2.2. Preparation of OFETs

The investigated transistors possessed bottom gate/top contacts architecture. The 100 nm thick Al bottom gate electrode was first deposited by physical vapor deposition (PVD) through a shadow mask on a cleaned glass substrate. This electrode was subsequently electrochemically oxidized in a buffered citric acid electrolyte solution to form a 30 nm thick Al_2_O_3_ barrier type gate dielectric. Various polymeric gate dielectrics—neat PVP, PVP: HMBG (4:1) and CEPVA—were deposited on top of the Al_2_O_3_ layer by the spin-coating method; from 5 wt % solution in PGMEA or DMF, respectively. All spin-cast films were preliminarily dried under vacuum (50 mbar) at 50 °C for 24 h and later subjected to various treatments and curing. Some CEPVA and PVP layers (further denoted as CEPVA50 and PVP50) were further dried under vacuum (50 mbar) at 50 °C for an additional 72 h. Samples denoted as CEPVA150 were prepared by additional curing of the CEPVA layer under vacuum (50 mbar) at 150 °C for 4h and then left to cool down in vacuum overnight. In the sample PVPCL the PVP layer deposited from PVP: HMBG (4:1) 5% solution was subsequently crosslinked. The crosslinking process was carried out under vacuum at 150 °C for 4 h.

The contact angle and surface roughness measurement of deposited thin CEPVA50, PVP50, CEPVA150, and PVPCL layers showed average value of contact angle; 70°, 67°, 69° and 76° and roughness; 12 Å, 8 Å, 13 Å and 9 Å (RMS, root mean square), respectively.

The TIPS-P active layer was deposited on the top of a polymer gate dielectric by dip-coating technique from 1 wt % solution in toluene. The samples were immersed vertically into the solution for 60 s and then pulled out at various withdrawing speeds ranging from 1 to 24 mm/min. The experiments were carried out at ambient laboratory conditions (temperature 21–24 °C and relative humidity = 30.0%−65.0%).

The deposition of the TIPS-P layers was precisely optimized to obtain the maximum charge carrier mobility of OFETs and to keep the same morphology of TIPS-P layers deposited on all the dielectric layers under study. The details of the deposition of the TIPS-P layers, and their morphology and mobility for various deposition rates can be found in the Supplementary Materials.

**Figure 1:**
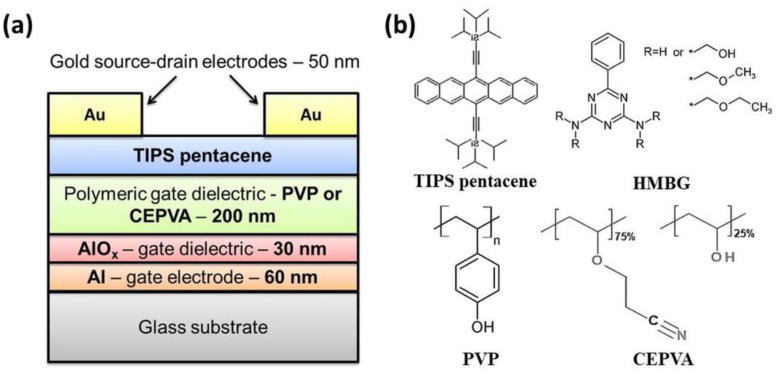
Scheme of the multilayer structure of the prepared organic field effect transistors (OFETs) (**a**) and chemical structure of used compounds (**b**).

As the last step, top 50 nm thick linear gold source and drain electrodes were deposited by physical vacuum deposition through a shadow mask. Due to the charge transport dominating along the a-axes of TIPS-P molecule the source-drain channel was oriented in the perpendicular direction with respect to the sample withdrawing direction during dip-coating, i.e., direction of the crystals formation [29,30,31]. Figure 1 shows a schematic drawing of the OFET and chemical structures of used organic materials.

### 2.3. Characterization

#### 2.3.1. Broadband Dielectric Spectroscopy (BDS)

Broadband dielectric spectroscopy (BDS) was performed on a few micrometer thick layers of CEPVA, PVP and PVP: HMBG polymers, respectively, (same as for the OFETs preparation) drop cast from solutions on Au-plated brass electrodes (diameter 20 mm). Prior to the measurements the samples were exposed first to 70 °C for 6 h in air and in vacuum at 70 °C for 6 h. The CEPVA samples were subsequently dried at 110 °C for 1 h in air, and finally for 4 h under vacuum at 110 °C. The PVP: HMBG (PVPCL) samples were cross-linked in the same manner as the thin layer used in the OFET (PVPCL). This slow drying process was necessary to avoid the formation of bubbles and nanovoids in the thick films. At the end the top gold electrode was deposited by PVD.

#### 2.3.2. Fourier-Transform Infrared Spectroscopy (FTIR)

Attenuated total reflectance (ATR) Fourier-transform infrared (FTIR) spectra of the CEPVA films cast on an Al foil were recorded in the range 4000–650 cm^−1^ at 256 scans per spectrum with 4 cm^−1^ resolution using a Nicolet Nexus 870 FTIR spectrometer (Thermo Electron Corporation, Madison, WI, USA). The samples were measured on an ATR Golden Gate unit with a diamond prism and a controlled heated top plate in a dry-air-purged environment. The films were heated from laboratory temperature (27 °C) up to 90 °C. The spectra were recorded at 5 °C steps in the intervals 30 °C–40 °C and 80 °C–90 °C and at 2 °C steps in the interval 40–80 °C, where structural changes were expected.

#### 2.3.3. Solid-State NMR

Variable-temperature solid-state NMR spectra were measured at 11.7 T using a Bruker AVANCE III HD WB/US NMR spectrometer (Karlsruhe, Germany, 2013) in a double-resonance 4-mm probe head at spinning frequencies ωr/2π = 10 kHz. The fine-powdered sample was placed inside a 4 mm ZrO_2_ rotor. ^1^H MAS NMR spectra were recorded using the single-pulse experiment with the repletion delay of 10 s and 256 scans. ^13^C MAS NMR spectra were recorded using the single-pulse experiment with a high-power dipolar decoupling (SPINAL-64). The applied short repetition delay (2 s) was used to suppress the ^13^C magnetization of rigid molecular segments and to enhance the signals of mobile units. The number of scans was 1024. A standard cross-polarization pulse sequence was used to record rigid molecular segments. The corresponding ^13^C CP/MAS NMR spectra were measured with the length of cross-polarization contact time of 500 µs. The applied nutation frequency of B1(^13^C) and B1(^1^H) fields during the cross-polarization period was 1/2 = 62.5 kHz and repetition delay was 5 s. During data acquisition the high-power dipolar decoupling SPINAL-64 was applied. The applied nutation frequency of B1(^1^H) field was ω1/2π = 89.3 kHz in this case. The number of scans was 1024. The spectra were measured in the temperature range of 20–80 °C. Compensation for frictional heating was taken into account.

#### 2.3.4. Wide-Angle X-Ray Scattering (WAXS)

Wide-angle X-ray scattering (WAXS) experiments were performed using a pinhole camera (MolMet, Rigaku, Japan, modified by SAXSLAB/Xenocs) attached to a microfocused X-ray beam generator (Rigaku MicroMax 003) operating at 50 kV and 0.6 mA (30 W). The camera was equipped with a vacuum version of Pilatus 300K 2-dimensional detector. Calibration of primary beam position and sample-to-detector distances was performed using Si powder sample. Experimental setups covered the scattering vector, q = (4π/λ)sinΘ, where λ is the wavelength and 2Θ is the scattering angle, in the range 0.22–1.42 Å^−1^. Homemade software based on PyFAI Python library (doi:10.1088/1742-6596/425/20/202012) was used for data reduction. Samples were measured in reflection mode with incident beam angle set to 1.3 degree.

#### 2.3.5. Surface Characterization

Thickness and roughness of prepared organic layers were examined with the use of surface profiler KLA TENCOR P-10 (KLA-Tencor, Milpitas, CA, USA). The effective thicknesses of the deposited electrodes, as well as the deposition rate were determined using a crystal balance monitor. The static contact angle of sessile drop was measured by a contact angle goniometer.

#### 2.3.6. OFET Electrical Characterization

The electrical characteristics were measured in an electrical circuit with a common grounded drain, using an electrometer Keithley K6517A (Keithley Instruments, Solon, OH, USA) in the gate branch and source-meter Keithley K2400 (Keithley Instruments, Solon, OH, USA) in the source-drain branch. Samples were investigated in air and in vacuum. For the electrical measurements in vacuum, the samples were placed in a vacuum chamber under the dynamic vacuum (pressure 10^−5^ mbar). The temperature dependences of the basic OFET electrical transfer and output characteristics were measured in the temperature interval between 10 and 70 °C. The measurements were done in quasi steady state conditions with temperature step 10 °C. The samples were left to equilibrate for 10 min at each step of the temperature cycling. Prior to the measurements, the samples were left in a high vacuum for about 3 days for degassing.

## 3. Results and Discussion

### 3.1. FTIR and Solid State NMR Characterization of the CEPVA Polymer 

In order to understand the possible impact of elevated temperature on CEPVA dielectric, and correlate it with the performance of OFETs, the FTIR analysis and solid state NMR study of CEPVA were performed in the temperature range of 20–90 °C. The FTIR analysis clearly indicates the presence of hydrogen bonds between residual hydroxyl groups of CEPVA polymer, which start to dissociate around 50 °C (see Figure S1). A similar effect was also observed in the not crosslinked PVP. 

At the same time the solid-state NMR studies revealed presence of labile physical network, which partially stabilize the CEPVA polymer even above its T_g_. However, above 50 °C this network became disintegrated and the mobility of both the polymer backbone and of cyanoethoxy side groups increased significantly. This finding correlates well with the results obtained from FTIR. The detailed analysis of FTIR and solid state NMR studies can be found in the Supplementary Materials. 

### 3.2. Broadband Dielectric Spectroscopy 

**Figure 2:**
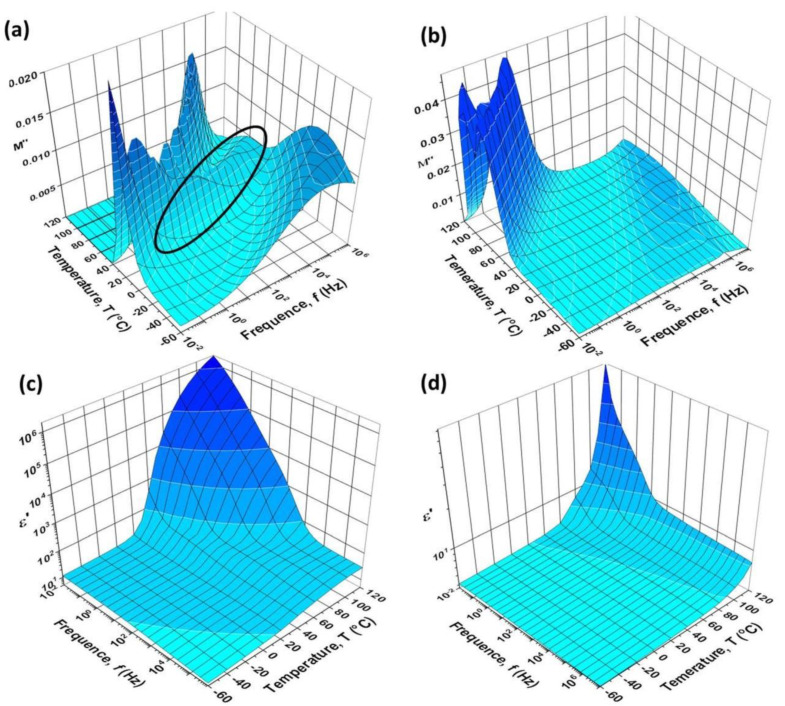
Dielectric spectra of CEPVA (**a,c**), with the relaxation band of the cyanoethyl side groups marked with the black circle, and PVP (**b,d**). Imaginary part of the loss modulus (**a,b**) and real part of permittivity (**c,d**). Measured in N_2_ atmosphere.

The real part of the permittivity (dielectric constant) at 1 Hz, obtained from the dielectric spectroscopy measured at room temperature, was ε’~13 for the CEPVA polymer, whereas the values ε’~5.5 and ε’~4 were found for PVP and PVPCL, respectively, which is in good agreement with previously reported data [32,33,34]. A difference in the dielectric constant between PVP and PVPCL arises from the decreased concentration of polar hydroxyl groups in the crosslinked samples due to their reaction with the crosslinking agent. It can serve as an indirect proof of crosslinking. Higher dielectric constant of CEPVA can be expected due to the presence of the highly polar cyanoethyl groups. The significant increase of its dielectric constant with increasing temperature, particularly at low frequencies, arises from the increasing mobility of cyanoethyl side groups above T_g_. The temperature dependence of the BDS spectra of the CEPVA polymer is shown in Figure 2 as the real part of the permittivity and the imaginary part of the loss modulus. In the investigated temperature range and frequencies, CEPVA shows mainly two kinds of relaxations. For the low frequency limit, the α-relaxation appears as a sharp peak at about 20 °C and the β-relaxation at −120 °C. Both relaxations shift to higher frequency with increasing temperature. An additional relaxation band (marked with the black circle in Figure 2) was observed between 30 and 100 °C, appearing at higher frequencies than the α-relaxation. This relaxation process was assigned to a relaxation of cyanoethyl side groups [35]. In the temperature range up to 40–50 °C this relaxation band was relatively narrow and intense. With increased temperature its intensity was reduced and the band was shifted to higher frequencies. Following the FTIR and NMR analyses, the evolution of the discussed relaxation could occur as a result of the hydrogen bonds dissociation at elevated temperature and cessation of their stabilization effect on the side groups and polymer main chain as well. 

### 3.3. Wide Angle X-Ray Scattering 

WAXS patterns acquired on the TIPS-P layers are in a good agreement with previously published data [36]. They showed a similar crystal structure of the TIPS-P for all the samples under study (see Figure S6). The mean size of the TIPS-P crystallites varied roughly between 26 and 52 nm, depending on which dielectric layer it had been deposited, but no correlation between the crystal size and charge carrier mobility was found within this crystal size range (see Supplementary Materials).

### 3.4. Performance of OFETs with Different Dielectrics 

**Figure 3:**
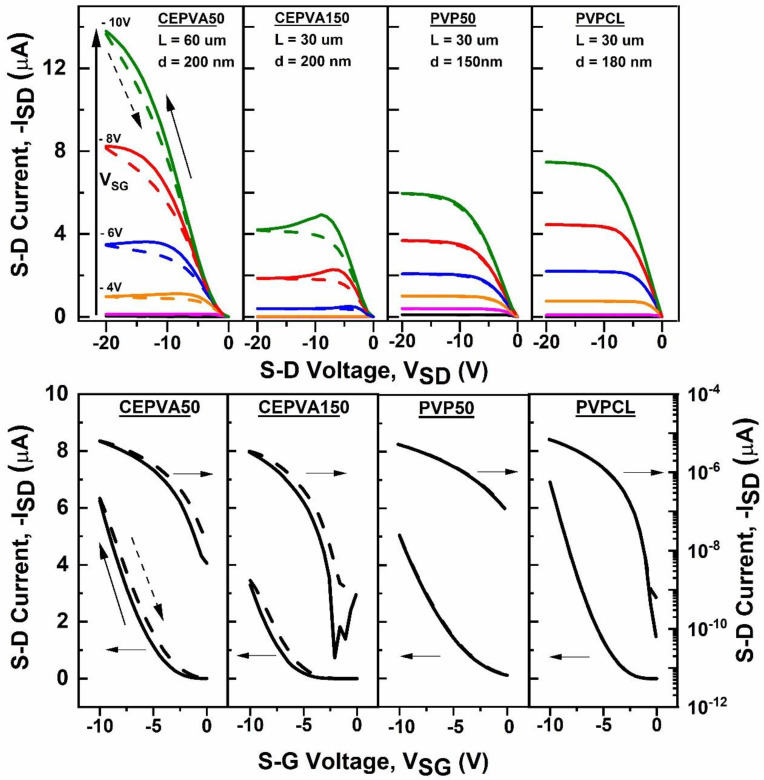
Output (top panel) and transfer (bottom panel, V_SD_ = −10 V) I–V characteristics of OFETs with different gate dielectrics. Measurement directions: solid line—increasing applied voltage and dash line—decreasing voltage. Measured in high vacuum (p < 10^−3^ Pa), gate sweep rate 1 s per full cycle.

**Figure 4:**
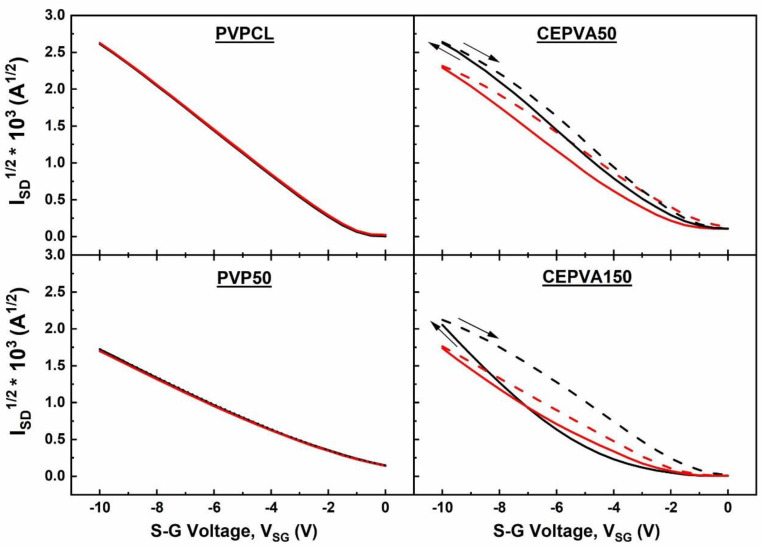
Square root of transfer characteristics measured at 30 °C for two different gate sweep rates - 50 s/cycle (black) and 1 s/cycle (red). Measurement direction: solid line—increasing voltage and dash line—decreasing voltage.

The transfer and output characteristics of the OFETs equipped with four different dielectric layers, i.e., CEPVA50, CEPVA150, PVP50 and PVPCL, respectively, are shown in Figure 3. Square root of transfer characteristics measured for two different gate sweep rates are shown in Figure 4. The samples were measured in vacuum at 30 °C in stabilized conditions after being kept for 72 h in a vacuum chamber under dynamic vacuum (10^−3^ Pa). In such conditions we could assume that the samples were free of moisture, residual solvents and with minimized oxygen content that could strongly affect the transistor performance as traps, dopants or by the charge–dipole interaction. The OFET with PVP50 dielectric exhibited almost ideal characteristics with only negligible hysteresis, even at the slow measurement rate (see Figure 4). This is in contradiction with the literature [23,37,38], where a marked hysteresis was observed on OFETs with the non-crosslinked PVP50, assigned to a slow orientation polarization of the polar groups of the polymer. However, since PVP is a relatively rigid material having a high T_g_ around 150 °C the slow-polarization based hysteresis described in the literature could arise from a small content of residual polar solvent or absorbed water. Our measurements were performed after the extended drying process (3 days at 50 °C and 3 days in high vacuum), when moisture and solvent-free conditions can be already achieved.

Although measured under the same conditions as with PVP50, the OFETs with CEPVA polymer dielectrics showed a marked hysteresis, with a higher current when the transfer characteristic was measured in the backward direction. The recorded I_SD_ was higher as the sweep rate was slower (see Figure 4). It is in accord with the low T_g_ of CEPVA and the relaxation bands of the loss modulus in the BDS spectrum for 30 °C shown in Figure 2.

#### 3.4.1. CEPVA Induced Spatial Inhomogeneity of Charge Carrier Mobility in OFET

It follows from Figure 3 and Figure 4, and Table 1 that there are also marked differences between the electrical characteristics of OFETs with CEPVA50 and CEPVA150 dielectrics. The OFET with the dielectric layer slowly dried at 50 °C showed higher currents and about 5 times higher mobility than the OFET with the dielectric fast dried at higher temperature. Both transistors showed a local maximum in the output characteristics when measured in the direction of increasing voltage, but in the case of CEPVA50 this maximum and at the same time the saturation regime were shifted towards higher V_SD_. For CEPVA150 this maximum was located near the pinch-off voltage V_SD_ = V_SG_ − V_th_, where the difference in the electric potential between gate and drain electrodes is close to 0 V. Although it is expected that the active channel region near the drain contact would be gradually depleted with increasing V_SD_ the width of the depletion region is negligible in comparison to the overall channel length. Consequently, the channel resistance integrated over the whole length of the active channel is essentially invariable and the current should not be affected.

Since the morphology and crystal structure of the semiconductor deposited on all tested dielectric layers were nearly the same it suggests that the differences in the dielectric layer imposed by different drying conditions have some impact on the charge carrier transport in the transistor channel. The observed phenomena can be explained using a theoretical model developed by Sworakowski et al., which assumes a gradient of the charge carrier mobility in the direction perpendicular to the interface with the dielectric, induced by the presence of dipoles at the surface of the dielectric layer [39,40]. Our experimental data obtained for OFETs with CEPVA150 closely follow the modeled characteristics when decreased charge carrier mobility close to the dielectric/semiconductor interface has been taken into account. According to this model the depletion of the transistor channel near the drain contact is equivalent to the decreased average channel thickness. In connection with the lower charge carrier mobility in the channel close to the interface, this leads to the observed decrease of I_SD_ current in the output characteristics. In frame of such a scenario the shift of the local maximum of the I_SD_ current towards higher V_SD_ voltages observed in the OFETs with CEPVA50 could be understood as follows:

(a) The hydrogen bond limited slow-orientational depolarization of the dielectric near the drain electrode leads to a time delayed channel depletion, which is equivalent to the time delayed reduction of the channel thickness.
(b) Since the active channel is actually thicker than it would arise from the potential difference between the drain and gate electrodes (due to the higher concentration of accumulated charges) the channel depletion shifts the pinch-off voltage to higher V_SD_ values.

In Figure 4, the hysteresis and shift of the threshold voltage are more pronounced at a slower sweep rate in the case of CEPVA150 having containing more mobile dipolar groups.

#### 3.4.2. Gate Voltage Dependent Charge Carrier Mobility

As the active channel thickness increases with increasing gate voltage the transfer characteristics start to deviate from the ideal behavior. As can be seen in Figure 4, in the OFETs with CEPVA150 dielectric this deviation at higher gate voltage is superlinear whereas for OFETs with CEPVA50 it has a sublinear character.

**Figure 5:**
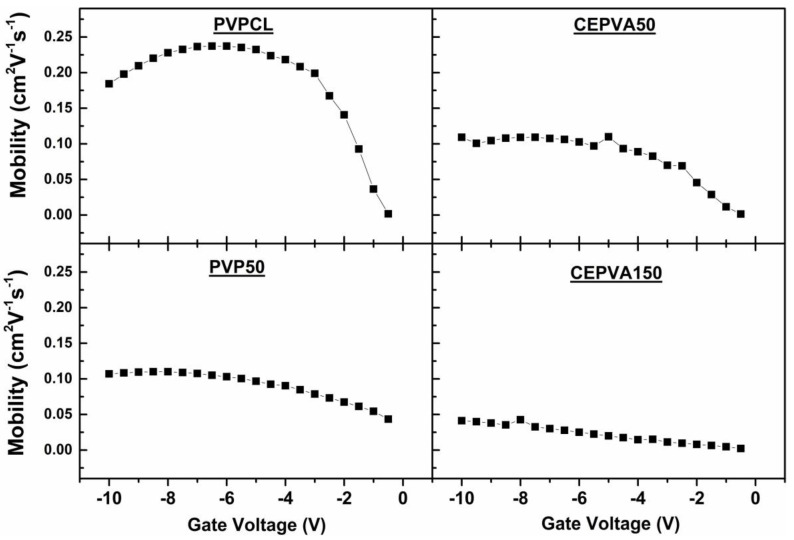
Dependence of charge carrier mobility on the applied gate voltage for OFETs with various kinds of dielectric layers.

In Figure 5 we plotted the dependence of the charge carrier mobility on the gate voltage, calculating the mobility using the equation commonly adopted in the literature:

(1) $$\mu_{sat}={\left(\frac{\partial \sqrt{I_{\mathrm{SD}\;\mathrm{sat}}}}{\partial V_{\mathrm{SG}}}\right)}^{2}\frac{2L}{\;WC}$$

It is obvious that contrary to many examples found in the literature [41,42,43], the mobility did not obey a simple polynomial dependence $\mu \;\sim \;{\left(V_{\mathrm{SG}}-V_{T}\right)}^{\gamma }$ and, hence, usual models adopted in the literature for data interpretation could not be applied.

Since the mean volume charge concentration at the bottom of the channel is $\frac{C^{2}{\left(V_{\mathrm{SG}}-V_{T}\right)}^{2}}{2kT\varepsilon_{S}}$ and the mean surface charge density is $C\left(V_{\mathrm{SG}}-V_{T}\right)$ [44] we get that the mean thickness of the conducting channel can be calculated from the equation:

(2) $$t_{\mathrm{con}}=\frac{2kT\varepsilon_{S}}{C\left(V_{\mathrm{SG}}-V_{T}\right)}$$

where C is capacitance of the gate dielectric layer and $\varepsilon_{S}$ relative permittivity of the semiconductor. The mean thickness calculated for higher gate voltages using the above equation is below 1 nm. Taking this into account we have to consider the following:

(a) The charge transport in such thin film OFETs is indeed a 1D process, controlled by the 1D drift–diffusion equation, where the charge mobility μ=μ(V(x)) is generally a function of the potential V(x). The analytically exact solution of this 1D drift–diffusion equation for general dependences μ=μ(V), and V=V(x) reported in ref. [44] shows that that Equation (1) is not fully correct. Therefore, the derivation of the FET mobility from the widely adopted equation
  (3) $$I_{\mathrm{SD}}\;\sim \;\mu_{\mathrm{FET}}\left(V_{\mathrm{SG}}-V_{T}-\frac{V_{\mathrm{SD}}}{2}\right)V_{\mathrm{SD}}$$
  is applicable only if the mobility is not dependent on the gate voltage. For other cases a different equation has to be used to calculate the mobility in the saturation regime of the OFET [44]:
  (4) $$\mu_{dG}=\left(\frac{\partial I_{SD}}{\partial V_{SG}}\right)\frac{L}{WC_{i}\left(V_{SG}-V_{th}\right)}$$
(b) Since mobile charges are accumulated in a very thin region near the semiconductor/dielectrics interface the dipoles present in the dielectric will have a strong impact on the energetic disorder of the semiconductor transport states. As the estimated thickness $t_{\mathrm{con}}$ is smaller or comparable with the space extent of the energy disorder, the approaches based on the 2D simulation of the FET characteristics using smoothly varying functions applied in the drift–diffusion equation, as used in various simulations (Silvaco, Comsol) are not very suitable. Instead, the charge transport is controlled by hopping, namely Marcus or Miller-Abrahams rates, between local segments carrying energy disorder. For the latter, usually the Gaussian disorder model is used. Depending on various technical details of the solution of the transport equation several approaches may be found in the literature [45,46,47,48,49]. All these approaches consider a randomized energy disorder in the total volume. The parameters of the Gaussian distribution of the transport states can be obtained from the experimental data using the relation [45]

(5) $$\mu =\mu_{0}exp\left(-{\left(\frac{2\sigma }{3kT}\right)}^{2}\right)exp\left[CF^{1/2}\left({\left(\frac{\sigma }{kT}\right)}^{2}-{\sum^{\text{​}}}^{2}\right)\right]$$

where *µ*_0_ means the charge carrier mobility in the absence of any energy disorder, σ and $\sum^{\text{​}}$ are the variances of the diagonal (local orbitals) and off-diagonal (transfer integrals) energy disorder.

#### 3.4.3. Impact of CEPVA Solidification on Dipolar Disorder

In searching for the nature of the charge carrier mobility gradient we should recall that the dielectric polarizability and the dipolar disorder could deteriorate the charge carrier transport in the adjacent semiconductor layer. In the OFETs with CEPVA150 gate dielectric layers, rapidly dried (over one night) at high temperature this effect can be more pronounced due to only partial formation of the hydrogen-bond network. Contrary to fast drying, a slow gradual removal of the residual solvent during a longer drying process at lower temperature (at 50 °C, low vacuum for 72 h for CEPVA50) yields enough time for the efficient hydrogen-bond network formation. The formation of hydrogen bonds is facilitated by a residual content of the polar solvent before the final film solidification. It slightly swells the polymer providing certain freedom of motion to the polymer side groups and allows for more favorable conformation of the backbone. Mentioned delayed depolarization is then understandable if we consider the limiting role of the hydrogen bonds network on the dielectric relaxation processes.

**Figure 6:**
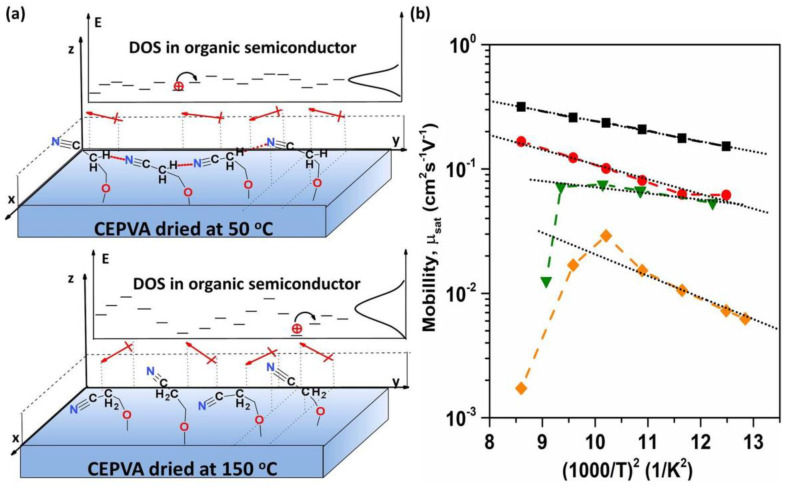
(**a**) Schematic representation of the CEPVA surface dipoles orientation in the *z* direction (perpendicular to the dielectric surface) after different drying regimes, and its impact on the DOS of the adjacent semiconductor layer. (**b**) Hole mobility µ_sat_ against square of reciprocal temperature for OFETs with various gate dielectrics. black—PVPCL, red—PVP50, green—CEPVA50, orange—CEPVA150.

However, not only hydrogen bonds formed between the residual hydroxyl groups can stabilize the polymer. As proposed in the case of cyanoethyl pullulan (CEP) by Xu et al. and in our previous study of CEPVA: PMMA blends, the cyanoethoxy side groups could be involved in a formation of hydrogen bonds between each other as well as with residual hydroxyl groups. In the CEP polymer the formation of hydrogen bonding between electronegative nitrogen and the adjacent electropositive α-H, –C≡N···H–C–C≡N was reported to facilitate a quasi-ordered association of the surface nitrile groups [27,28,50]. In the latter case, almost the same dependence of the OFET charge carrier mobility on the drying conditions of CEP used as the gate dielectric was reported as in our work. It suggests that similar associates are present at the surface of CEPVA50, too. Such quasi-ordering can significantly decrease the static disorder of surface dipoles (see Figure 6a). In the OFETs with more disordered surface dipoles (fast dried dielectric layer) the local charge–dipole interactions result in a broadened distribution of semiconductor DOS, making it less favorable for the hopping transport of charges.

We plotted the mobility against 1000/T^2^ in Figure 6b. The Pool-Frenkel-like second exponential term in Equation (5) could be neglected due to the low electric field applied in our case. First, we calculated the mobility from the linear part of the I_SD_^1/2^ transfer characteristic at the saturation region using Equation (1). For CEPVA based gate dielectrics we used the capacitance C_i_ taken from the temperature dependent dielectric constant in Figure 2. As seen in Figure 6b, both the PVP based samples, crosslinked and not crosslinked, exhibited good linear dependences in the whole temperature region, with σ = 0.06 eV and 0.07 eV for PVPCL and PVP50, respectively. In the OFETs with CEPVA based dielectrics two different regions of the mobility dependence on temperature could be distinguished: with positive temperature coefficient dµ/dT > 0 at low temperatures and negative temperature coefficient dµ/dT < 0 (Figure 6b) at temperatures above 50 °C. This indicates that for temperatures below ca 50 °C, the level of the energy disorder is constant. Upon reaching some critical temperature, the energy disorder σ will significantly increase with temperature. The term $exp\left(-{\left(\frac{2\sigma }{3kT}\right)}^{2}\right)$ in Equation (5) will then decrease with the temperature and the mobility becomes significantly lower. Our estimates show that the energy disorder increases to values of about 0.3 eV. 

The temperature interval 50–60 °C, where the mobility abruptly decreases coincides with the temperature, in which segmental motion in CEPVA is released and hydrogen bonds network is disintegrated. As the temperature increases, the system is changing to a highly viscous liquid, which makes fitting according to the Equation (5) at higher temperature impossible. Fitting the linear region of dµ/dT > 0 gave  σ values markedly higher for CEPVA150 but, on the contrary, it was slightly lower for CEPVA50 than for PVP based OFETs (see Table 1). Since there was a deviation from the linearity observed in many transfer characteristics we took into account the gate-dependent mobility extracted from the transfer characteristics using Equation (4) and calculated µ_dG_ from the temperature dependences of the mobility obtained for various V_SG_ (see Table 1). The overall smaller values of obtained for the highest V_SG_ reflect narrowed transport states distribution due traps filling at higher charge carrier concentration.

Besides the OFET with the crosslinked PVPCL dielectric there was a small permanent decrease in the mobility observed after the heating/cooling cycle for all other OFETs under study. This deterioration of the charge carrier transport during temperature cycling was attributed to not a full recovery of the hydrogen bonds network. It follows the idea of the broadening of the semiconductor DOS distribution by local charge–dipole interactions due to the increase in dipolar disorder close to the interface. It has been shown previously that these interactions can modify the local polarization energy within first few layers of the semiconductor layer [24,25,26]. This effect also corresponds to the decrease of mobility and to the increase of the local energy disorder with increasing dielectric constant of the polymer dielectrics used in these studies.

Despite of this trend, there was an exception observed in the case of CEPVA50, which, although having the same dielectric constant as CEPVA150, yields OFET with much better electrical characteristics and almost the same µ and σ as the OFET with significantly less polar PVP50 dielectric (see Table 1). It could be understood on the basis of well-preserved hydrogen bonds and a formation of quasi-ordered associations of surface nitrile groups in CEPVA50 as a result of drying conditions. As a consequence, the semiconductor in the OFET with CEPVA50 gate dielectric shows (i) much higher charge carrier mobility, (ii) lower local energy disorder, σ = 0.045 eV, (iii) weaker dependence of σ on the gate voltage and (iv) lower threshold voltage (see Table 1).

**Figure 7:**
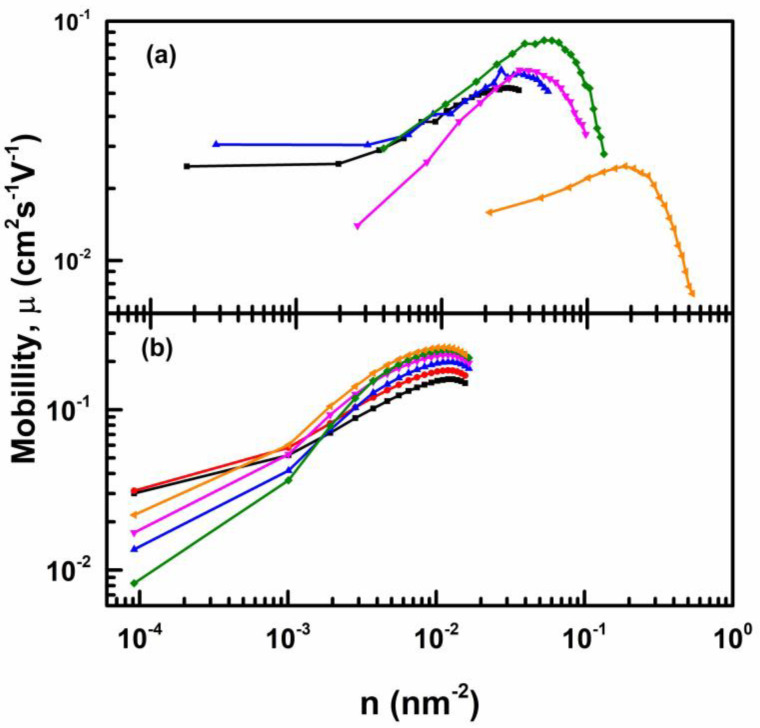
The dependence of the hole mobility on the surface charge concentration in the TIPS-P layer deposited on top of (**a**) CEPVA50 and (**b**) PVPCL for various temperatures: 10 °C (black), 20 °C (red), 30 °C (blue), 40 °C (pink), 50 °C (green) and 60 °C (orange).

OFET with CEPVA50 also shows a linear dependence of I_SD_^1/2^ on V_SG_ over wider range of the gate voltage compared to the superlinear dependence observed on OFET with CEPVA150 (see Figure 4). The small sublinear deviation observed at the high V_SG_ limit for the OFETs with the CEPVA50 and PVPCL dielectrics could arise from the decreased density of unoccupied “fast” states available for hopping transport at very high charge concentration and relatively low energy disorder. Such dependence of the mobility on the charge concentration was also predicted in the model published by Toman et al. for OFETs with P3HT active channel [49]. Although the cited model was originally developed for conjugated polymers it could be also applied for our case since the anisotropy of the charge transport, dominating along the a-axis of TIPS-P molecules, and the mobility around 0.1–0.3 cm^2^ s^−1^ V^−1^ were similar. In Figure 7 we plotted the dependence of the gate dependent mobility (Equation (4)) on the charge carrier concentration calculated using the relation N_h_ = C (V_SG_−V_th_). We found that the charge mobility increased with increasing charge concentration (i.e., with increasing gate voltage), which has been assigned to the trap filling in the disordered systems. We also see that upon reaching a critical charge concentration the mobility saturated or even decreased as the overlap between occupied and unoccupied states decreased. The dependences shown in Figure 7 were controlled by the level of the energy disorder *σ* as it has been theoretically demonstrated previously by the mobility dependences on the charge concentration (i.e., on the Fermi level) [46,47,48,49]. Interestingly, similar dependences as in Figure 7 were also found for the dependence of the diffusion coefficient of photogenerated polarons on their concentration in P3HT [51]. Due to higher dielectric constant at elevated temperature the markedly higher charge concentration was reached within the range of applied V_SG_ voltage for OFETs with CEPVA50, particularly at temperatures above 50 °C. At higher temperatures the mobility decreases also began at higher by the level of the energy disorder charge concentration. It can be understood if we consider an extended distribution of DOS when the hydrogen bonds network is disintegrated. Namely, we should take into account that for temperatures of ca 60 °C, the energy disorder was ca σ=0.3 eV.

## 4. Conclusions

The presence of the hydrogen bonds stabilization network in a high-k dielectric layer of cyanoethylated PVA (CEPVA) was proved by IR and NMR spectroscopy. It was shown that such network, particularly the formation of a quasi-ordered structure of dipolar groups at the interface with the adjacent semiconductor, had a significant impact on the charge transport in the conducting channel of the OFET. It resulted particularly in a higher mobility due to the reduced local energy disorder and, hence, narrower distribution of hopping sites.

It was shown that the hydrogen bonds formation could be influenced by the conditions during the layer formation and solidification—slow drying of the layer cast from the solution allowed for more extended hydrogen bonds network. In OFETs containing high-k polymer dielectrics with a low T_g_, as in the case of CEPVA, not stabilized polar side groups generally deteriorated the transistor performance due to the charge–dipole interactions. Since these interactions got weaker with the charge–dipole distance, the width of the DOS distribution and hence the mobility of charges changed with increasing distance from the semiconductor/dielectric interface. Since the effective thickness of the conducting channel in OFETs with high-k dielectrics might be in the order or below 1 nm the dipoles present in the dielectrics had a major impact on the energetic disorder of the transport states in the semiconductor and, hence, on the charge transport. It also had an impact on the shape of the OFET characteristics and on the dependence of the charge mobility on the gate voltage. Moreover, the impact of dielectric dipoles was manifested by an apparent decrease of the charge carrier mobility with increased temperature. This behavior solely arose from the increased intensity of charge–dipole interactions caused by the significant increase of CEPVA polarizability at higher temperature due to thermodynamic changes of the polymer. It clearly followed that it was very important to analyze the temperature dependence of mobility extracted from OFET measurements in frame of the thermal stability of the dielectric layer.

The tunability of the dielectric constant of the CEPVA polymer with temperature can be also utilized to characterize the charge transport over the wide range of charge carrier concentrations.

## Figures and Tables

**Table 1:** Comparison of charge carrier mobility, µ_sat_, threshold voltage, V_th_, and local energy disorder, σ, of the gate voltage dependent, µ_sat_ = f(V_SG_), and gate voltage independent mobility, µ_sat_ ≠ f(V_SG_), for investigated OFETs with different dielectric layers prepared under different drying conditions.

Sample	ε (1 Hz, 30 °C)	µ_sat_ ≠ f(V_SG_) (cm^2^ V^−1^s^−1^) at 30 °C		V_th_ (V) at 30 °C		σ (eV) from µ_sat_ ≠ f(V_SG_)		σ _dG_ (eV) from µ_sat_ = f(V_SG_)		
		Before Heating	After Cooling	Before Heating	After Cooling			V_SG_ = −4 V	V_SG_ = −7 V	V_SG_ = −10 V
PVPCL	4	0.22	0.21	−1	−1.5	0.055		0.07	0.06	0.05
PVP50	5	0.11	0.08	−0.5	−1.5	0.07		0.08	0.07	0.06
CEPVA50	21	0.10	0.06	−1.5	−1.5	0.045	T > 55 °C ~ 0.3	0.05	0.03	~0
CEPVA150	21	0.02	0.01	−4	−2	0.10		0.11	0.10	0.09

## Supplementary Materials

The following Supplementary Materials are available online at https://www.mdpi.com/2073-4360/12/4/826/s1, Figure S1: Evolution of FTIR spectra of CEPVA and PVP with temperature, Figure S2 and Figure S3: Solid state variable-temperature 1H MAS NMR and 13C CP/MAS + 13C MAS NMR spectra of CEPVA, Figure S4: Optical microscopy images of TIPS-P layer deposited by dip-coating with various deposition speeds, Figure S5: (a), (b) UV-vis absorption spectra of TIPS-P deposited with various deposition speed with unpolarized and linearly polarized light, respectively, (c) Range of charge carrier mobility in the OFETs with CEPVA50 dielectric and TIPS-P layer deposited at various speed, Figure S6: Wide-angle X-ray scattering profiles of thin films of TIPS-P deposited on four various dielectric surfaces.
