# Supplementary material for: Impact of Hydrogen Bonds Limited Dipolar Disorder in High-k Polymer Gate Dielectric on Charge Carrier Transport in OFET
Source: Polymers (Basel). 2020 Apr 5;12(4):826. doi: 10.3390/polym12040826 (PMC7240453; doi:10.3390/polym12040826)
Supplement: Supplementary file 1 [file polymers-12-00826-s001.pdf]

Supporting Information

Impact of hydrogen bonds limited dipolar disorder in high-k polymer gate dielectric on charge carrier transport in OFET

Bartosz Paruzel,* Jiří Pfleger, Jiří Brus, Miroslav Menšík, Francesco Piana, Udit Acharya

Institute of Macromolecular Chemistry, Academy of Sciences of the Czech Republic, 162 06 Prague 6, Czech Republic

* Correspondence: paruzel@imc.cas.cz (B.P)

1. Fourier-transform infrared (FTIR) spectra of CEPVA and PVP

The vibration spectrum of CEPVA (Figure S1) shows two bands at 2924 and 2882 cm^{-1}, which were assigned to the asymmetric and symmetric stretching vibrations of $–CH_2–$ aliphatic groups, respectively. The band at 1479 cm^{-1}, assigned to scissoring vibrations, was also linked to the $-CH_2-$ group. The band at 1358 cm^{-1} was connected to the CH group. The most intensive band at 1091 cm^{-1} was attributed to the stretching vibration of the (C–O–C) ester group. The band of nitrile stretching vibration was observed at 2250 cm^{-1}.

Besides the typical bands of the poly(2-cyanoethyl vinyl ether), there was also a broad band between 3700 and 3200 cm^{-1} with a maximum at 3500 cm^{-1} and a shoulder at 3350 cm^{-1} typical for stretching vibrations of hydroxyl groups. The presence of the hydroxyl groups arose from a not complete cyanoethylation of PVA, the substitution being only about 80%. With increasing temperature the intensity of the shoulder on the lower wavenumbers side decreased and the maximum of the band slightly shifted towards higher wavenumbers. The position of other bands in the spectrum remained virtually unchanged during heating.

The shape of the OH absorption band at lower temperatures pointed to the presence of the H-bonded hydroxyl groups, which are known to absorb in the range 3550–3230 cm^{-1}. Its narrowing upon heating corresponded to an increasing fraction of free –OH groups with their typical absorption in the region 3670–3580 cm^{-1}. Small changes in the absorption band intensity can be explained by a variation of the mechanical contact between the ATR crystal and sample due to the change in the elasticity of the polymer.

There was a difference between the behavior of CEPVA50, in which the broad band of the hydroxyl group reached the maximum intensity at about 54 °C, and CEPVA150 with maximum observed at about 62 °C. In both cases, the intensity of the bands at 2924 and 2882 cm^{-1} assigned to the $–CH_2–$ group followed the same course. The bands of other vibrations remained unchanged, which confirms that curing at higher temperature does not bring any chemical changes to the polymer structure.

The FTIR spectra of PVP can be described according to the literature as follows: The bands at 2920 and 2846 cm^{-1} can be assigned to the stretching vibration of the $–CH_2–$ groups, the band at 3020 cm^{-1} to the stretching vibration of the CH group on the aromatic ring, the doublet at 1611 and 1596 cm^{-1} together with the sharp band at 1509 cm^{-1} to the stretching vibration of the C=C bond in the aromatic ring, the bands at 1170 cm^{-1}, 1100 cm^{-1} and 1014 cm^{-1} and 824 cm^{-1} to the deformation vibration of the aromatic rings, two bands at 1443 and 1220 cm^{-1} are connected to the O-H deformation vibration combined with C-O stretching vibration, and the band with maximum at 3300 cm^{-1} is assigned to the stretching vibration of the –OH in phenol group. The maximum of the latter band shifts with thermal treatment, reaching the final position 3348 cm^{-1} at 90 °C. Similarly as discussed in the case of CEPVA, this shift could be explained by the dissociation of hydrogen bonds of the hydroxyl groups. However, the difference of the intensities during heating was smaller.

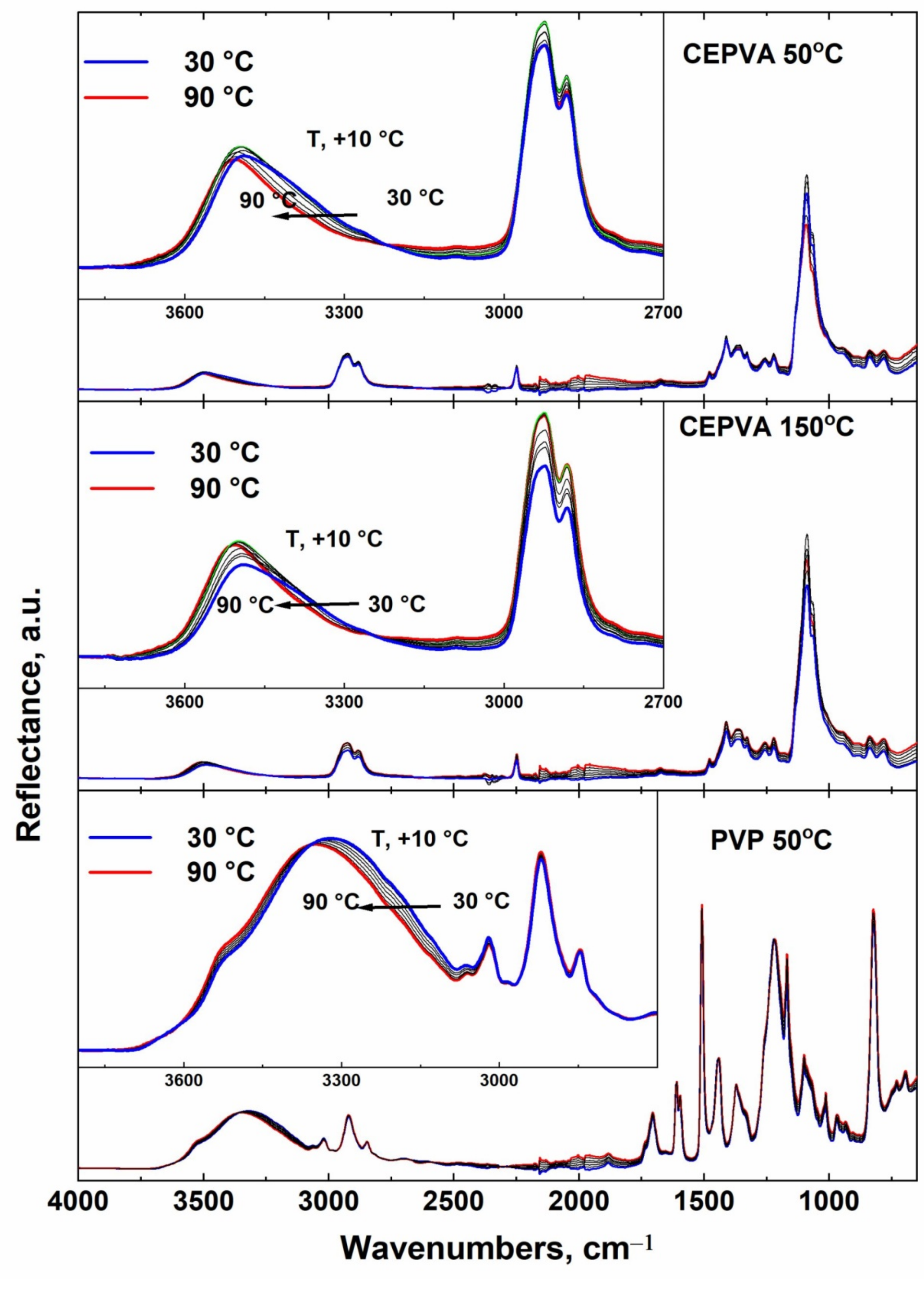

Figure S1. FTIR spectra of CEPVA50 (top), CEPVA150 (middle) and PVP50 (bottom) polymer dielectrics. Inset—evolution of hydroxyl group vibration band with temperature.

2. Solid state NMR of CEPVA

2.1 ^{1}H MAS NMR—global dynamics.

The ^1H MAS NMR spectra (see Figure S2) measured at variable temperature can provide information about global segmental dynamics of the investigated systems. Typically, broad featureless ^1H MAS NMR signals accompanied by strong spinning sidebands (ssb's) indicate rigid organic solids in the crystalline or amorphous state, which were below or close to the glass transition temperature (T_g). Narrowing of the signals towards the linewidth of ca. 2 kHz–500 Hz and the disappearance of ssb's indicate partially released motions typical for amorphous solids above T_g and near the critical fusion temperature T_c. The resolved ^1H MAS spectra with the signals ca. 500-50 Hz reflect soft amorphous phase or gels with significantly enhanced segmental motions, whereas diluted solutions or liquids produce narrowed signals below 50 Hz.

In the case of the investigated CEPVA system, the ^1H MAS NMR spectrum recorded at 295 K was featureless accompanied by the strong spinning side-bands indicating restricted segmental dynamics. All polymer segments (i.e., main chains as well as side chains) were rigid adopting similar motions. At 315 K the spinning side bands disappeared, which corresponded quite well to low T_g of CEPVA being around 300 K [1,2]. This process was accompanied by the slight narrowing of the central signal. An additional narrow signal appeared at ca. 2.5 ppm reflecting a fraction of CH_2 groups with considerably released motions.

As reflected by the broad and narrow spectral lines, the investigated system in the temperature range from 315 to 340 K consists of the two components. With increasing temperature the amount of the rigid fraction (reflected by the broad spectral component) gradually decreased. Above 340 K the system consists of the single relatively mobile (soft) component.

Although in the temperature region from 315 to 340 K segmental motions were gradually released, liquid like behavior was not reached up to ca. 340 K. As the recorded signals did not show typical narrowing, the system did not adopt completely isotropic high frequency and high amplitude motions. Rather the system resembles gel-like materials in which polymer chains persisted in partially crosslinked arrangement. Above 345 K the recorded signals considerably narrow indicating relatively free high-amplitude and high-frequency nearly isotropic motions.

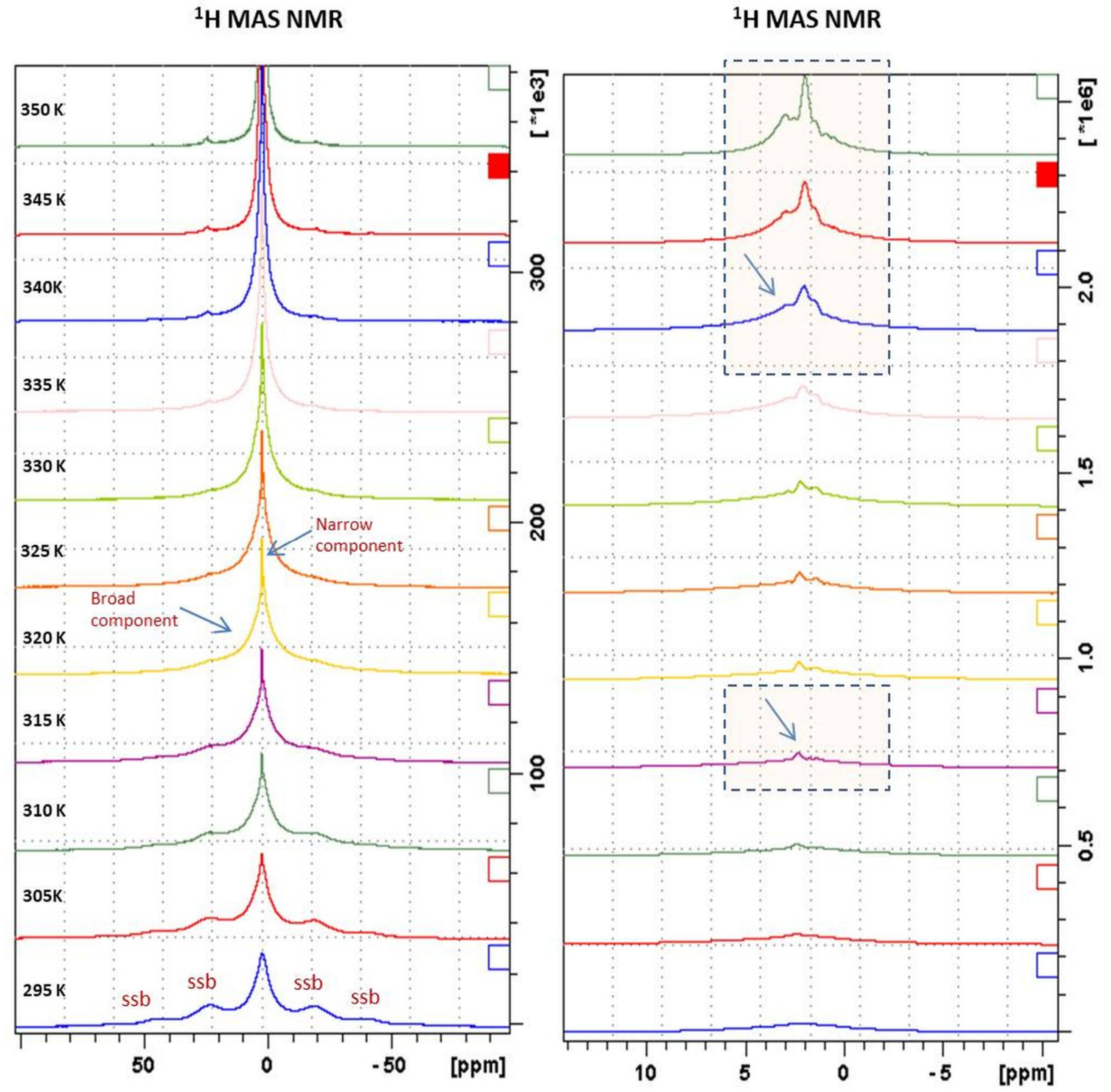

Figure S2. Variable-temperature ^{1}H MAS NMR spectra of CEPVA: a) full spectral range and b) expanded spectral region.

2.2 ^{13}C MAS AND CP/MAS NMR spectroscopy—site-specific dynamics.

In contrast to ^{1}H MAS NMR spectroscopy the ^{13}C solid-state NMR spectra due the considerably enhanced spectral resolution provide site specific information as the individual signals can be directly attributed to the corresponding molecular segments. Specifically for CEPVA (Figure S3) the side-chain units CN (#1) resonated at ca. 120 ppm, while the side-chains units CH_2 (#2) and CH_2-O (#3) resonated at ca. 65 and 30 ppm, respectively. The backbone units CH-O (#5) and CH_2-O(#4) resonated at ca. 85 and 40 ppm, respectively.

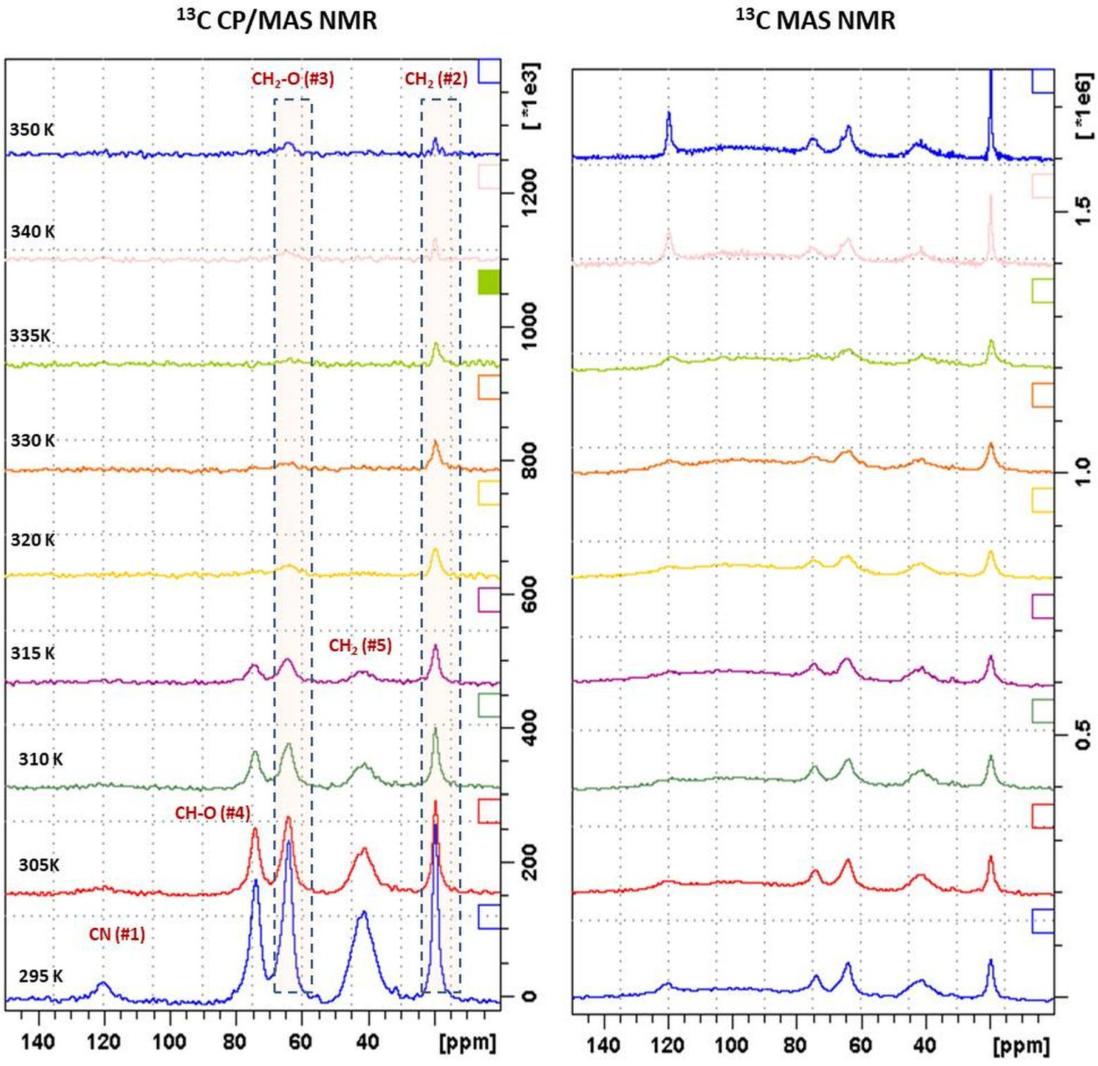

Figure S3. Variable-temperature ^{13}C CP/MAS (left) and ^{13}C MAS NMR (right) spectra of CEPVA, respectively.

The two-component character of the investigated CEPVA system was clearly demonstrated in the recorded solid-state ^{13}C NMR spectra. Whereas the ^{13}C CP/MAS NMR spectra selectively detected rigid components, the single-pulse ^{13}C MAS NMR spectra measured at a short repetition delay (1-2 s) instead detected mobile fractions. For CEPVA at 295 K the rigid fraction clearly predominated as reflected by the corresponding ^{13}C CP/MAS NMR spectrum (Figure S3, left) in which strong signals were easily detected. In contrast, a certain fraction of partially mobile polymer chains also existed in the systems at this temperature as reflected by the signals detected in the corresponding single-pulse ^{13}C MAS NMR spectrum (Figure S3, right). Molecular segments of this fraction, however, still executed low-amplitude motions, which is indicated by broadening of the corresponding signals.

With increasing temperature, the ^{13}C CP/MAS NMR signals rapidly disappeared. Above 315 K the weak signals of side-chain CH_2 units at 30 and 65 ppm could be detected only, while the main-chain signals were not effectively excited due to the released segmental motions. This finding indicates that the rigid fraction was transformed to the soft mobile fraction as the segmental motions are released. However, the motional amplitudes were still relatively low because the signals in the corresponding ^{13}C MAS NMR

spectra did not exhibit significant narrowing. Consequently, we expected a weak physical network in this temperature range. With high probability the crosslinks of this physical network were provided by a small fraction of side-groups, which could interact via hydrogen bonding, existence of which was confirmed by means of FTIR spectroscopy (see the previous section). The same trend in the intensity development in this temperature range for various peaks in FTIR spectrum of CEPVA also suggests a relatively strong stabilization effect of hydrogen bonds network on the overall polymer structure. This bonding seems to be dynamic reversible as indicated by the broadening of the corresponding ^{13}C NMR signal at ca. 120 ppm and by evolution of hydroxyl group absorption band in FTIR spectrum of CEPVA cured at 150 °C. Interestingly, the rapid decrease in the signals intensity in ^{13}C CP/MAS NMR spectra was not accompanied by the increase in signals intensity in the corresponding ^{13}C MAS NMR spectra. Moreover, at 330 K all signals in the ^{13}C MAS NMR spectrum nearly disappeared. This phenomenon could be explained by the destructive interference of segmental motions, where the frequency was comparable with the frequency of dipolar decoupling being ca. 80-100 kHz. With an additional temperature increase above 340 K this gray zone of solid-state NMR spectroscopy was overcome and the narrow high-intensive signals were clearly detected in the corresponding ^{13}C MAS NMR spectra. This finding indicates that the crosslinks were disintegrated and the physical network disappeared, the system was transformed to the highly viscous liquid.

3. Optimization of the TIPS-P layer deposition process—dependence of charge carrier mobility (OFET) and UV-Vis absorption on the deposition rate

Figure S4 shows crossed polarization optical microscopy images of TIPS-P layers deposited on the CEPVA surface by dip-coating at different withdrawing speeds. In all cases we could see good homogeneity of crystals as well as the space between them, which suggest uniformity of cast layers. The formation of elongated crystal structures (parallel to withdrawing direction) was clearly seen on layers cast at all deposition speeds. For higher speeds, 16 and 24 mm/min, the formation of an irregular bunch of elongated crystals was visible. When the withdrawing speed decreased longer, thicker, more regular and well packed, needle-like crystals occurred. At the lowest speed 1 mm/min, very well ordered and almost perfectly aligned, however significantly wider crystals were formed. Additionally, some platelet crystals were found in few spots created probably due to the broadening and interconnection of elongated crystals. It can be explained by a negligible meniscus formation during withdrawing of the substrate from the solution at such low speed and thus a very small concentration gradient [3]. Formation of platelet crystals could be explained by a misalignment of TIPS-P molecules condensed on already formed crystal. Such a decrease of layer anisotropy was pronounced as an increase of the absorption of linearly polarized light perpendicular (at 100° or 280°) to the sample withdrawing direction for the samples deposited at the slowest withdrawing speeds, as seen in Figure S5b. A molecular disorder in preferential direction can influence the charge carrier transport.

For direct comparison of the influence of the used polymer dielectrics on the morphology of the TIPS-P layer, the deposition on PVP and on CEPVA was performed at the same conditions. We obtained the same or very similar needle-like crystal morphology of TIPS-pentacene layer in all cases. It could be expected due to the similarity of surface energy and surface roughness in the all three kinds of dielectric layers. The highest charge carrier mobility was obtained for TIPS-pentacene thin films cast at a speed of 4 mm/min.

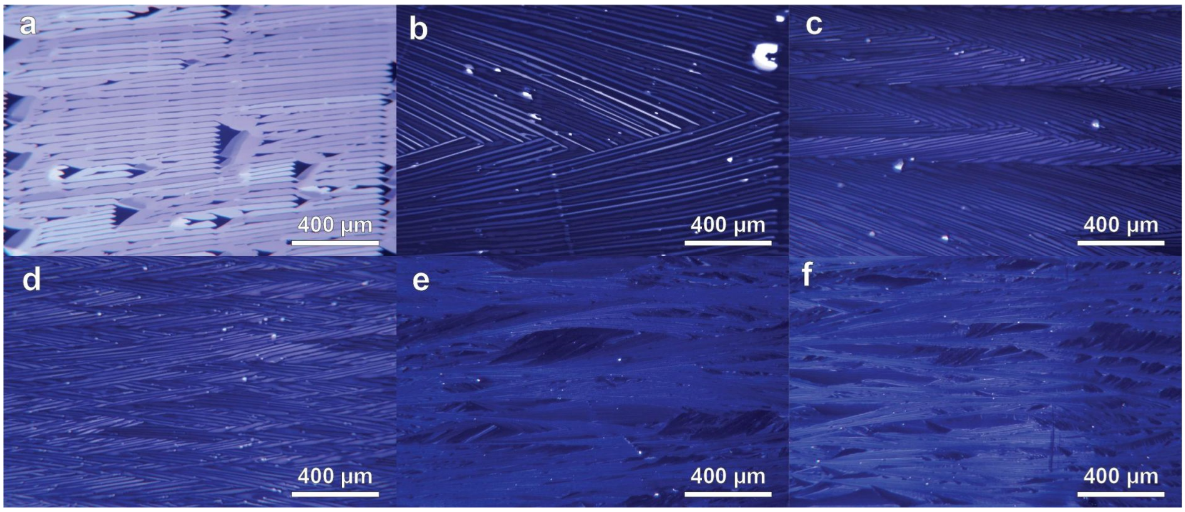

Figure S4. Optical microscopy images with crossed polarizers of samples dip-coated with six withdrawal speeds: a) 1 mm/min; b) 4 mm/min; c) 8 mm/min; d) 12 mm/min; e) 16 mm/min and f) 24 mm/min.

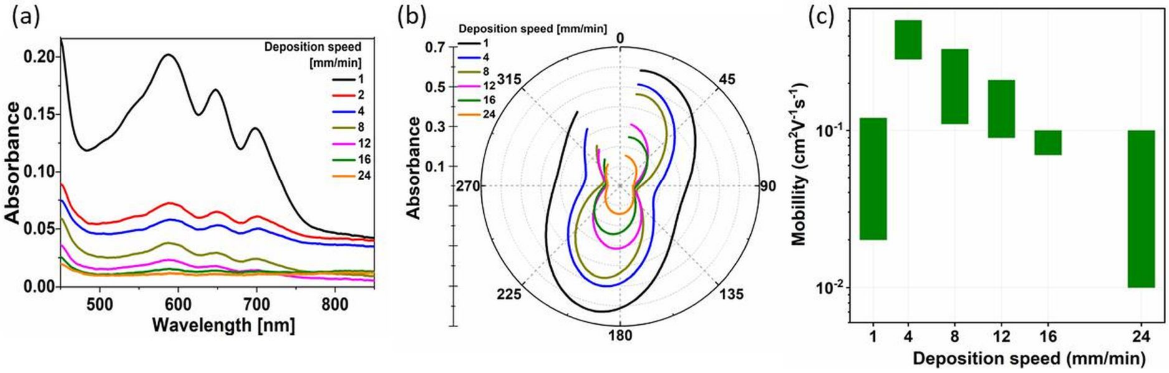

Figure S5. (a) UV-vis spectroscopy with unpolarized light. (b) Dependence of the absorbance of linearly polarized light (λ = 648 nm) on the polarization direction. The withdrawal direction is parallel to 10° axis. (c) Range of charge carrier mobility in the CEPVA50 OFETs for various deposition rates of the TIPS-P layer. Measured in the ambient conditions.

Due to needle-like crystallite morphology of the cast TIPS-P layers and due to the charge transport dominating along the a-axes of TIPS-P molecules, the anisotropy of the charge transport can be expected. For this reason the top gold source and drain electrodes were deposited in the direction making the transistor channel perpendicular to the crystals axis of the semiconductor [3–5]. In Figure S5c the dependence of the mobility measured in ambient conditions on the withdrawing speed for OFETs with CEPVA50 dielectric are shown, based on the testing more than 30 transistors for each speed. All of these transistors exhibited a very low threshold voltage (close to zero), the current ON/OFF ratio higher than 10^3 and significant hysteresis in the transfer characteristics. The observed increase of the mobility with decreasing speed of casting up to 4 mm/min could be easily understood base on the improvement of anisotropy of the crystalline morphology and closer crystals packing. The decrease of the mobility for layers deposited at the slowest speed seemed unexpected but it can be explained if we consider (i) isotropic character of platelet crystals grown in the lowest deposition speed, which work as a charge transport limiting bridges between the needle-like crystallites, and (ii) significant increase in the width of these needle-like crystals in case of samples prepared at 1 mm/min, compared to 4 mm/min and higher

speeds, which suggests increased probability of molecular disorder, which was actually confirmed by smaller anisotropy observed by polarized UV-VIS absorption.

4. Wide-angle X-ray scattering.

WAXS measurements of the TIPS-P layers are showing similar crystal structure for all the samples under study, independently on the polymer dielectric used in OFET (see Figure S6). The obtained peak positions are in a good agreement with published data [6]

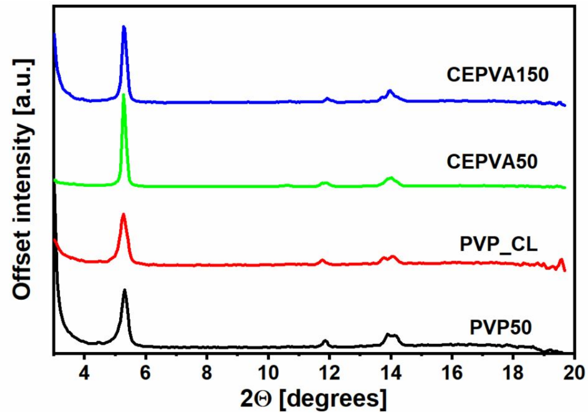

Figure S6. Wide-angle X-ray scattering profiles of thin films of TIPS-P deposited on four various dielectric surfaces with sample withdrawing speed 4 mm/min

Almost perfectly flat background indicates almost 100% crystallinity of prepared layers. The mean size of crystallites was calculated based on the reflection at 2Θ = 5.3° using the Scherrer equation:

$$L = \frac{K\lambda}{\beta \cdot cos\Theta} \quad (1)$$

where K is the shape factor and β is the full width at half maximum of the reflection (in radians). The mean sizes of the crystallites calculated for the thin film of TIPS-P deposited on the surface of CEPVA150, CEPVA50, PVP50 and PVPCL using the same deposition speed were 42.0, 52.8, 26.5 and 28.0 nm, respectively. The largest crystallites were found in the sample CEPVA50, where the crystallites penetrate almost the whole thickness of the TIPS-P film.

References

1. Piana, F.; Kredatusová, J.; Paruzel, B.; Pfleger, J. Polymer blends of poly(2-cyanoethyl vinyl ether) and poly(methyl methacrylate) with improved dielectric properties for flexible electronics. *Express Polym. Lett.* **2017**, *11*, 731–737. https://doi.org/10.3144/expresspolymlett.2017.70.
2. Piana, F.; Pfleger, J.; Jambor, R.; Řičica, T.; Macak, J.M. High-k dielectric composites of poly(2-cyanoethyl vinyl ether) and barium titanate for flexible electronics. *J. Appl. Polym. Sci.* n/a-n/a. https://doi.org/10.1002/app.45236.
3. Sele, C.W.; Kjellander, B.K.C.; Niesen, B.; Thornton, M.J.; van der Putten, J.B.P.H.; Myny, K.; Wondergem, H.J.; Moser, A.; Resel, R.; van Breemen, A.J.J.M.; et al. Controlled Deposition of Highly Ordered Soluble Acene Thin Films: Effect of Morphology and Crystal Orientation on Transistor Performance. *Adv. Mater.* **2009**, *21*, 4926–4931. https://doi.org/10.1002/adma.200901548.
4. Haddon, R.C.; Chi, X.; Itkis, M.E.; Anthony, J.E.; Eaton, D.L.; Siegrist, T.; Mattheus, C.C.; Palstra, T.T.M. Band Electronic Structure of One- and Two-Dimensional Pentacene Molecular Crystals. *J. Phys. Chem. B* **2002**, *106*, 8288–8292. https://doi.org/10.1021/jp0207937.

5. Ostroverkhova, O.; Cooke, D.G.; Hegmann, F.A.; Tykwinski, R.R.; Parkin, S.R.; Anthony, J.E. Anisotropy of transient photoconductivity in functionalized pentacene single crystals. *Appl. Phys. Lett.* **2006**, *89*, 192113. https://doi.org/10.1063/1.2387135.
6. Feng, L.; Tang, W.; Zhao, J.; Yang, R.; Hu, W.; Li, Q.; Wang, R.; Guo, X. Unencapsulated Air-stable Organic Field Effect Transistor by All Solution Processes for Low Power Vapor Sensing. *Sci. Rep.* **2016**, *6*, 20671. https://doi.org/10.1038/srep20671.
